# Recurrent pregnancy loss, psychological distress and wellbeing support for women: a mixed-methods analysis

**DOI:** 10.1186/s12905-025-04079-2

**Published:** 2025-11-03

**Authors:** Áine Aventin, Martin Robinson, Jonathan White, Martina Galeotti

**Affiliations:** 1 School of Nursing & Midwifery, Belfast, Northern Ireland United Kingdom; 2 School of Psychology, Belfast, Northern Ireland United Kingdom; 3 Early Pregnancy and Recurrent Pregnancy Loss Service, Northern Health and Social Care Trust, Coleraine, Northern Ireland United Kingdom

**Keywords:** Health & wellbeing, Women’s health, Maternal mental health, Perinatal mental health, Miscarriage, Recurrent miscarriage, Recurrent pregnancy loss

## Abstract

**Background:**

Research suggests multiple experiences of miscarriage, also known as recurrent pregnancy loss (RPL), may be associated with compounded risk for adverse mental health impacts. However, there is a need for research evidence that offers clarity in relation to potential predictors of psychological distress and wellbeing among this neglected population of women. This paper aims to explore physical and experiential factors associated with miscarriage-related psychological distress and wellbeing among women who had experienced RPL.

**Methods:**

Adopting a mixed methods approach, the paper presents a secondary analysis of data which assessed the perceived impact of miscarriage experience as measured by the Revised Impact of Miscarriage Scale (RIMS) in a cross-sectional survey of (*n* = 839) women in Northern Ireland and thematic analysis of qualitative interviews with a sub-sample of the women (*n* = 10) who experienced RPL.

**Results:**

Quantitative analysis indicated that women who experienced two or more losses reported significantly greater miscarriage-related distress when compared with those who experienced one miscarriage. This was true for the ‘Devastating Event’ and ‘Loss of Baby’ RIMS subscales, however, differences between groups related to ‘Isolation and Guilt’ were non-significant. Qualitative analysis identified themes consistent with the RIMS. These included potential predictors of distress such as devastating memories, anticipated loss and disenfranchised grief, as well as feelings of isolation and guilt stemming from social stigma and inadequate support.

**Conclusion:**

The experience of RPL is extremely distressing, with the potential to lead to maternal morbidity. To support women’s reproductive health and rights, compassionate healthcare that includes mental health and memory-making support are important during miscarriage and subsequent pregnancies.

**Supplementary Information:**

The online version contains supplementary material available at 10.1186/s12905-025-04079-2.

## Background

Miscarriage is defined as the involuntary loss of an embryo or foetus before it can survive independently (i.e. before 20- or 24-weeks’ gestation depending on regional definition). Affecting around 15% of clinically recognised pregnancies, the estimated population prevalence of recurrent pregnancy loss (RPL) defined as two or more losses, is 2.6% [1]. While several physical and environmental predictors of RPL, including chromosomal, anatomical and auto-immune abnormalities, have been documented, it is reported that more than 50% of losses are due to unknown causes [2].

Research indicates that miscarriage can be associated with increased distress and psychopathology for both women and men [3, 4]. Evidence suggests that while for most people distress reduces over time, psychological difficulties can remain a concern up to 9-months following miscarriage [1, 5]. Women who experience miscarriage are 1.5 times more likely to report a mental health difficulty (e.g. depression, anxiety, adjustment disorder) at 12-month follow-up relative those who do not experience miscarriage [6]. Among risk factors for adverse psychological health outcomes are lack of adequate support, experience of loss at increased gestational age, the recency of loss experience, and the experience of multiple pregnancy losses [3, 5].

Comparative studies have suggested that women who report RPL experience significantly greater psychological distress and poorer quality of life relative to those without miscarriage experiences and those who report a single miscarriage experience [2, 6, 7]. Previous evidence has suggested that the number of pregnancy losses experienced may confer additive risk, i.e. a greater number of miscarriages is associated with more mood- and anxiety-related distress [8]. Further research which examines the potential for additive risk is needed, however, as findings in relation to this have been inconsistent. Some studies have found non-significant differences in mental health outcomes between groups of women experiencing one or more losses [9]. A recent systematic review and meta-analysis of the mental health impact of perinatal loss concluded that, due to the limited numbers of studies included, further exploration of the impact of RPL is necessary [10].

Variables such as gestational age may also be considered as relevant predictors of wellbeing outcomes following pregnancy loss. For example, it has been suggested that parent-infant attachment increases with gestational age, hence, loss at a later stage of pregnancy results in significantly greater post-loss distress [11]. This is supported by previous evidence which found that self-reported pre-natal attachment and timing of pregnancy loss is significantly associated with grief and depression outcomes, suggesting that women who report greater attachment and loss at a later term experience greater psychological distress [12]. This is supported by qualitative research with parents who experienced RPL in which women reported significant anxiety about subsequent pregnancy outcomes and reduced emotional attachment was used as a coping strategy during pregnancy to mitigate anticipated distress [13].

With more than 50% of RPLs resulting from unknown aetiology, it is paramount that practitioners seek to reduce the psychological impact of these losses on women. Understanding the potential impact of physical and experiential variables on women’s distress following RPL can help identify women at higher risk of psychological distress and inform the development of appropriate healthcare support. This paper aims to investigate physical and experiential factors associated with miscarriage-related psychological distress and wellbeing among a sample of women who have experienced RPL.

## Methodology

### Design

This paper reports a secondary analysis of data from a primary study which aimed to understand the healthcare experiences and emotional needs of women who attended a hospital in Northern Ireland (NI) for treatment during miscarriage [14]. The primary study took place between January 2020 and April 2023. It adopted a sequential mixed-methods approach, combining a scoping review of the international literature [14] with analysis of self-reported cross-sectional online survey data from 723 women who had experienced miscarriage, 20 semi-structured interviews with women, and written narrative accounts from 24 health professionals in NI. A study advisory group comprising 5 members (nurse gynaecologist, bereavement midwife and two female service users) consulted on the co-creation of study materials and recruitment and dissemination processes. Ethical approval was obtained from the Queen’s University Belfast Faculty of Medicine, Health and Life Sciences Research Ethics Committee on 10th December 2021 (Ref: MHLS 20_99).

## Quantitative methods

### Survey materials

Pregnancy loss variables were recorded using a bespoke battery of questions assessing: the number of miscarriages experienced, and details related to most recent miscarriage experience such as time passed since this experience, number of weeks gestational age at the time miscarriage, and the method of conception for this pregnancy. A breakdown of the online survey is provided in Additional File 1.

Distress associated with pregnancy loss experiences was measured using the Revised Impact of Miscarriage Scale (RIMS) [15]. The RIMS measures transdiagnostic indicators of miscarriage-related distress across three domains:

- Isolation and guilt: Explores feelings of self-blame, inadequacy, and being misunderstood by others.
- Loss of baby: Assesses the personal sense of loss and the mourning process for the expected child.
- Devastating event: Measures the perceived severity of the miscarriage as a life-altering and disruptive experience.

The scale is used to measure the experiences of miscarriage and not as a diagnostic tool. Responses are recorded on a 4-point Likert scale from 4 “Definitely true for me” to 1 “Definitely not true for me” indicating agreement with 16 statements indicative of distress related to miscarriage experiences. Responses are summed to produce a summary score for each subscale and overall indictor of miscarriage-related distress, with greater scores indicative of worse distress. Possible score range are: 5–20 for the *Loss of Baby* and *Devastating Event* subscales, 6–24 for the *Isolation and Guilt* subscale, and 16–64 for the total scale. Internal reliability was favourable for the total RIMS (Cronbach’s α = 0.91), and for each constituent subscale (Loss of Baby, α = 0.85; Devastating Event, α = 0.82; Isolation and Guilt, α = 0.81).

### Survey sample & recruitment

The survey sample size for the primary study was calculated using a formula to estimate unknown parameters from a target population in cross-sectional studies using a random sample [16]. Although there is no baseline prevalence of miscarriage in the study population in NI (miscarriage figures are not routinely collected), it has been estimated that annually, between 10 and 20% of clinical pregnancies end in miscarriage (*N* = 3000–5000) [17]. Using a formula [16] to apply the estimated range of 10–20% for the phenomenon of miscarriage, indicated a minimum required sample of between 138 and 240.

The final sample was much higher than anticipated with a total of 839 respondents comprised of women living in NI who had experienced a miscarriage (i.e. loss of a pregnancy before 24 weeks gestational age) in the previous five years. While the quantitative analysis presented in the primary study adopted a complete case approach including *n =* 723 women who responded to all survey questions, the current analysis adopted an available case approach based on the *n* = 839 women who provided responses to the primary outcome measure in this analysis (RIMS). Women were recruited via advertisements on social media (Facebook, Instagram, Twitter) which provided information on the study and a direct link to the survey. The advertisements were shared by the research team among their professional and personal contacts and network. Although several pregnancy loss charities were approached to support recruitment, only the UK pregnancy loss charity ‘SANDS’ shared the survey link on its Northern Irish Facebook page. Further, upon request from the research team, nine different closed Facebook peer-led pregnancy loss groups agreed to share the advertisement in their communities.

Participants were recruited based on the following criteria:

- Inclusion criteria:
  - Women who experienced miscarriage and attended a hospital setting in Northern Ireland in the last five years
  - Women who were 16+ years old at the time of survey completion.
- Exclusion criteria:
  - Women who did not live in Northern Ireland at the time of their miscarriage.

### Quantitative data collection

Quantitative data were collected with an internet-based cross-sectional survey January - April 2021. Respondents accessed the survey via social media advertisements and completed anonymised questionnaires using Qualtrics software. Participants were not offered incentives.

### Quantitative data analysis

Quantitative data analysis was conducted by MR. The data were found to violate assumptions of normality, therefore non-parametric inferential statistics were used. Testing study hypotheses, we examined the association of miscarriage experiences (time passed since loss, gestational age at time of loss, number of pregnancy losses) and distress (RIMS domains: Loss of baby, Isolation and guilt, and Devastating event) using Spearman’s Rank correlations, an effect size coefficient ranging from − 1 to 1 indicating the strength of correlation between non-normally distributed variables [18]. A threshold for significant associations of *p* <.05 for further assessment using Kruskal-Wallis tests and Dwass-Steel-Critchlow-Fligner (DSCF) pairwise comparisons. These analyses included analysis of Epsilon-squared (ε²) as a measure of effect size to facilitate comparison of independent variables’ association with outcomes. This effect size measure ranges from 0 to 1 with greater values indicative of more substantial effect [18]. All quantitative analyses were performed using Jamovi [19] and presented in Additional File 2.

## Qualitative methods

### Interview materials

Informed by the quantitative findings, an interview guide for women who had experienced miscarriage aimed to explore their experience and establish their perceptions of what, if anything, should be changed to help meet their emotional support needs in hospital settings. Questions posed to respondents probed reflection on emotional needs post-miscarriage (e.g. “*Do you have any suggestions about how your emotional needs might have been better supported in hospital settings*,* while you were experiencing your miscarriage?*”). The interview topic guide is provided in Additional File 3.

### Interview participants & recruitment

At the end of the survey, respondents were invited to provide their email address if they were interested in receiving more information about participating in a semi-structured interview. If they were interested, they clicked on a link which took them to a separate form. A total of 302 women left their email address and a random selection of 20 were selected (using a random number generator) to take part in the primary study. These women were sent an information sheet and consent form and asked to email the researcher if they still wished to take part. Of the 20 women who took part in the primary study, 10 had experienced more than one miscarriage and are included in the current analysis.

### Qualitative data collection

Semi-structured interviews were conducted online by MG using Microsoft Teams with 20 women between October 2021-February 2022. Only the data provided by those who experienced RPL (two or more miscarriages) (*n* = 10) were utilised for the current analysis. Participants were not offered incentives.

### Qualitative data analysis

Qualitative data analysis was conducted by ÁA using verbatim transcriptions of interview audio recordings from the primary study [14]. The transcripts were imported to NVivo 12 for coding. To preserve confidentiality, participants were allocated a unique identifier code, for example, ‘P1’ to represent Participant 1. Interview transcripts were read several times to immerse the researcher in the data. As the aim of the qualitative analysis was to explore in detail, factors contributing to or ameliorating distress by women experiencing RPL, the data were initially categorised using the three RIMS headings (‘Loss of Baby, ‘Isolation and Guilt’ and ‘Devastating Event’) as overarching categories. During this initial process it became evident that an additional overarching category ‘Wellbeing Supports’ was needed to enable organisation of all data. Following this categorisation using a deductive process, thematic analysis and open inductive coding was carried out following the Braun and Clarke [20] framework. Following analysis, two co-authors (ÁA and MG) discussed coding, theme labels and definitions, and agreed representative participant quotes to illustrate themes.

## Results

### Sample descriptives

The majority of survey respondents were aged 31–39 (*n* = 420, 58.09%) and lived with a partner and children (*n* = 507, 70.22%). A plurality held bachelors-level qualifications (*n* = 298, 41.22%), and most held professional/managerial employment (*n* = 370, 51.18%).

At the time of survey most women reported experiencing miscarriage 1–2 years previous (*n* = 279, 33.25%) or 3–4 years previous (*n* = 237, 28.25%). The majority reported single miscarriage experience, i.e. one pregnancy ending miscarriage in their lifetime (*n* = 489, 58.28%), and natural conception of most recent pregnancy ending in miscarriage (*n* = 800, 95.35%). Of those who reported RPL most reported a history of two miscarriages (*n* = 184, 21.93%), followed by three (*n* = 87, 10.37%), and four or more (*n* = 79, 9.42%). Sample demographic characteristics are summarised in Table 1.

**Table 1:** Demographic characteristics of online survey sample

Characteristic	*n* (%)
*Age Bracket*	
16–25	48 (6.64)
26–30	156 (21.58)
31–39	420 (58.09)
40–49	99 (13.69)
*Living Status*	
Alone	23 (3.19)
With partner	191 (26.45)
With partner and other children	507 (70.22)
With partner and other people	1 (0.14)
*Highest Level of Qualification*	
No formal qualification	3 (0.41)
Standard grade/GCSE	64 (8.85)
Higher/A-Levels	93 (12.86)
College	95 (13.14)
BSc Degree	298 (41.22)
Higher degree	170 (23.51)
*Time Since Most Recent Miscarriage*	
Less than a year ago	199 (23.72)
1–2 years ago	279 (33.25)
3–4 year ago	237 (28.25)
5 + years ago	124 (14.78)
*Conception Method of Most Recent Miscarriage*	
Natural conception	800 (95.35)
Assisted reproductive therapies	39 (4.65)
*Number of Miscarriages*	
1	489 (58.28)
2	184 (21.93)
3	87 (10.37)
4+	79 (9.42)
*Gestational Age of Most Recent Miscarriage*	
Less than 4 weeks	6 (0.72)
Between 4 and 6 weeks	113 (13.47)
Between 7 and 12 weeks	583 (69.49)
Between 13 and 16 weeks	65 (7.75)
Between 17 and 20 weeks	46 (5.48)
Between 21 and 24 weeks	24 (2.86)
Unknown	2 (0.24)

Percentage reported correspond to proportion of non-missing responses

Miscarriage-related distress measured by the RIMS was found to be positively skewed across domains, i.e. a majority of women reported increased levels of distress across all subscales. The average score was greatest for sense of ‘Isolation and Guilt’ (Range = 6–24, M = 18.68, Mdn = 19, IQR = 16–22), followed by ‘Devastating Event’ (Range = 6–20, M = 17.68, Mdn = 19, IQR = 16–20), and ‘Loss of Baby’ (Range = 5–20, M = 17.29, Mdn = 18, IQR = 16–20). The total score summing RIMS items had a potential range of 16–64 and was similarly found to be positively skewed in this sample (M = 53.65, Mdn = 56, IQR = 49–60).

For the qualitative analyses (see Table 2), the ten participating women all had experienced two or more miscarriages (range = 2–6). No further demographic information was gathered from the women.

**Table 2:** Interview participant ID and number of miscarriages

Participant ID	Number of Miscarriages
P1	4
P2	2
P3	3
P4	2
P5	2
P6	3
P7	2
P8	6
P9	3
P10	4

### Quantitative analysis

Outcome variables (RIMS total and subscales) were found to be correlated with the number of pregnancy losses reported and gestational age of most recent pregnancy loss with small effect sizes for these associations [21], see highlighted cells in Table 3. Both these clinical variables were positively correlated with RIMS scores, i.e. an increased number of pregnancies ending in loss and increased gestational age of most recent pregnancy loss were associated with higher scores on each of the RIMS domains, suggesting greater distress experienced. The recency of latest miscarriage experience was not significantly correlated with RIMS domains.

**Table 3:** Correlation matrix of associations between miscarriage experience variables and outcomes

	1		2		3		4	
1. Number of Miscarriages	—							
2. Time Since Miscarriage	.07	(.050)	—					
3. Gestational Age	.13	(<.001)	.01	(.666)	—			
4. RIMS Total	.19	(<.001)	- .01	(.707)	.13	(<.001)	—	
5. Devastating Event	.20	(<.001)	- .04	(.222)	.12	(<.001)	.80	(<.001)
6. Loss of Baby	.16	(<.001)	<- .01	(.932)	.19	(<.001)	.82	(<.001)
7. Isolation and Guilt	.16	(<.001)	.01	(.880)	.09	(.010)	.91	(<.001)

Spearman’s rho correlation coefficient reported to two decimal places (*p*-value reported to three decimal places). Matrix column headings correspond to variable numbers specified

Associations between outcome variables, number of miscarriages, and gestational age of most recent miscarriage were thus further explored using Kruskal-Wallis tests (see Table 4). The effect sizes observed for association between these were found to be small [21], and the most pronounced effects observed were number of pregnancy losses on feelings of experiencing a Devastating Event (ε² = 0.041, *p* <.001), and gestational age on Loss of Baby (ε² = 0.056, *p* <.001).

**Table 4:** Results of Kruskal-Wallis tests comparing association between miscarriage experience and distress

Outcome	Number of Miscarriages					Gestational Age				
	χ²	*p*-value	df	ε²	Pairwise Contrasts^a^	χ²	*p*-value	df	ε²	Pairwise Contrasts^b^
Devastating event	34.61	< 0.001	3	0.041	1 < 2 (< 0.001) 1 < 3 (< 0.001) 1 < 4+ (0.002)	17.58	0.007	6	0.021	4–6 < 17–20 (0.022) 7–12 < 17–20 (0.027)
Loss of baby	21.29	< 0.001	3	0.025	1 < 2 (0.009) 1 < 3 (0.006) 1 < 4+ (0.024)	47.23	< 0.001	6	0.056	< 4 < 17–20 (0.035) 4–6 < 17–20 (< 0.001) 4–6 < 21–24 (0.004) 7–12 < 17–20 (< 0.001) 7–12 < 21–24 (0.003) 13–16 < 17–20 (0.025)
Isolation and guilt	22.79	< 0.001	3	0.027	1 < 2 (0.011) 1 < 3 (0.013) 1 < 4+ (0.003)	12.17	0.058	6	0.015	-
RIMS Total	30.14	< 0.001	3	0.036	1 < 2 (0.001) 1 < 3 (0.001) 1 < 4+ (0.001)	23.27	< 0.001	6	0.028	4–6 < 17–20 (0.002) 7–12 < 17–20 (0.001)

χ² = Chi-quare, *df* = Degrees of Freedom, ε² = Effect size (Epsilon-squared)

^a^ Categorical comparison of number of pregnancies ending in miscarriage (1, 2, 3, 4+). Significant contrasts (*p*-values reported to three decimal places)

^b^ Categorical comparison of weeks gestational age of most recent miscarriage (Unknown, < 4, 4–6, 7–12, 13–16, 17–20, 21–24). Significant contrasts (*p*-values reported to three decimal places)

Pairwise comparisons revealed that those who reported two, three, and more than four miscarriages reported greater miscarriage-related distress relative to those who reported experiencing a single miscarriage. Pairwise contrasts between recurrent loss groups however suggested that the distress reported in each outcome domain did not differ between those who reported two, three, and four or more miscarriages (see Additional File 2).

Pairwise contrasts assessing the effects of gestational age at time of loss highlighted differences on feelings of experiencing a Devastating Event, Isolation and Guilt, and RIMS Total score between those who experienced a miscarriage later in their pregnancy (17–20 weeks) relative to earlier (4–6 weeks, 7–12 weeks). Further differences were noted in Loss of Baby feelings and RIMS total score between those reporting miscarriage experience in later gestational age categories, once again with older gestational age at time of miscarriage associated with increased reporting of distress (see Table 4). Results of all pairwise contrasts are presented in Additional File 2).

### Qualitative analysis

**Fig. 1:**
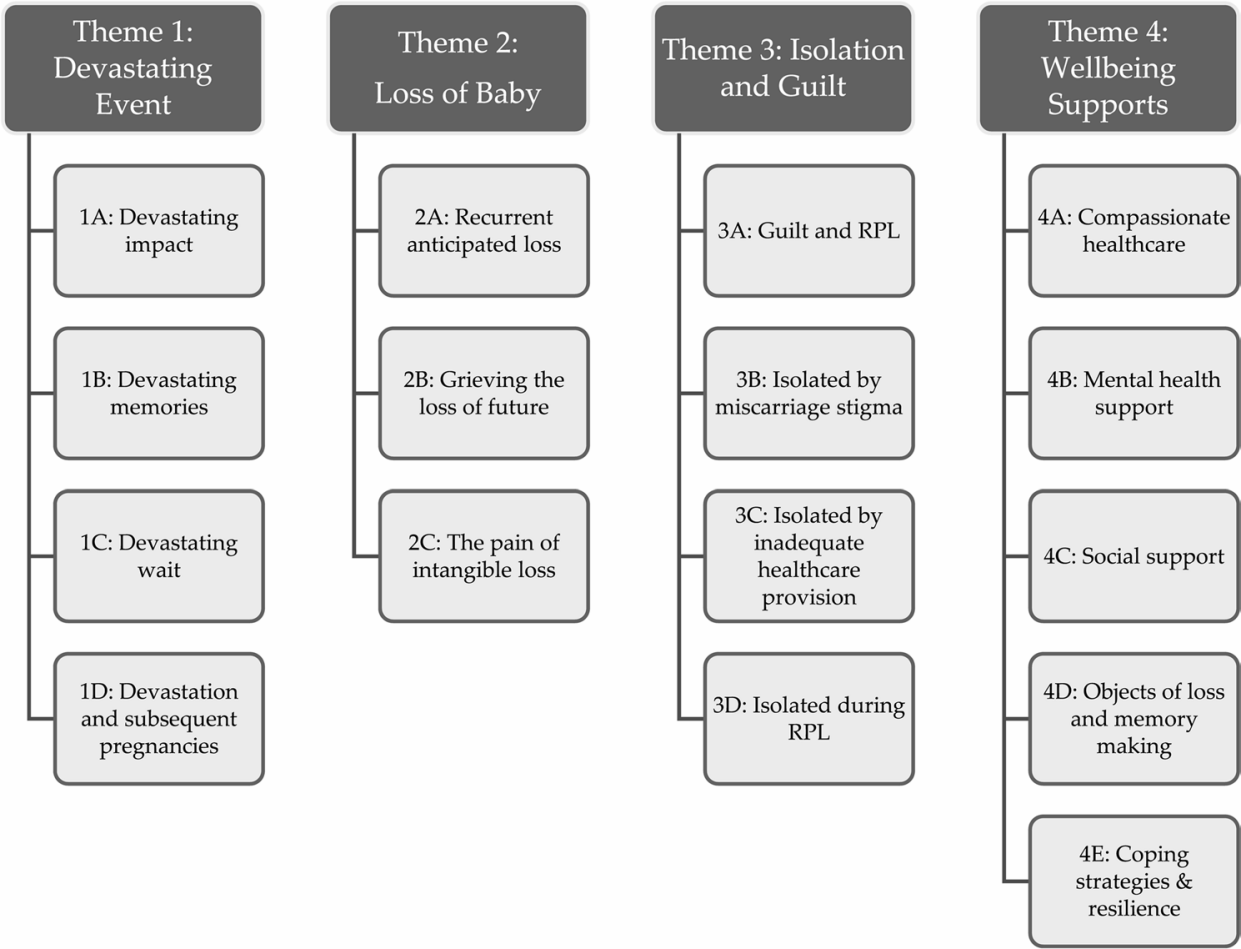
Themes and sub-themes identified from analysis of interview data

The themes and sub-themes identified through analysis of women’s semi-structured interview responses are illustrated in Fig. 1 and additional illustrative quotes are provided in Additional File 4.

### Theme 1: devastating event

#### “Over and over and over”: the devastating impact of RPL

Most participants talked about the devastating impact of their RPL experiences. This was illustrated by descriptions of the distressing and often shocking impact of receiving the news of loss, as well as the longer-term impacts on their mental health.

Several women described the distress they experienced on hearing the news of another loss:

*“And this happens over and over and over. Every pregnancy*,* every miscarriage*,* and even when you are pregnant. You’re on tenterhooks*,* your*,* your*,* your… your nerves is away.” P8*.

For some, their miscarriages were followed by mental health problems including anxiety, depression and post-traumatic stress symptoms:

*“I developed severe anxiety […] I didn’t want to leave the house. I was crying all the time.” P2*.

*“I don’t feel well mentally because in your head you’re going over the pictures of everything that the scenario*,* the scenes.” P8*.

Some women also talked about the devastating impact on their husbands:

*“[My husband] was very upset*,* you know*,* he was*,* he was crying. It was the first time I’ve ever seen him cry.” P9*.

#### “Full-on-labour” and “The same room”: devastating memories

Women described devastating memories of the physical experiences of their miscarriages and the treatment they received. Several women described the intensity of the physical experience, which they felt unprepared for:

*“I had a full-on labour*,* very intense physical experience which I was completely unprepared for (participant starts to cry).” P4*.

Many of the women shared memories of insensitive care that added to their feelings of devastation:

*“The miscarriage itself was devastating but the way that I was spoken to about it*,* I think it was nearly worse. It was like the child I had didn’t matter.” P3*.

Several women remarked on the devastation of being treated in the same room in which they had experienced previous losses. It was as though the memory of being treated in that room previously, somehow added to their feelings of devastation.

*“You are brought back into the same room over and over again so you’re like re-living what happened the first time*,* you know it was quite difficult.” P10*.

#### “Mentally you’re not ready for that”: a devastating wait

For women experiencing RPL, waiting for confirmation or completion of miscarriage was devastating, especially when they knew from previous experience what the likely outcome would be. Some women spoke of the wait in the hospital while they were actively miscarrying:

*“I waited a good 7 hours. And the worst thing was*,* the worst few hours of that was because my cervix was open*,* and I was so scared that the baby was gonna fall out.” P1*.

Others spoke of the devastation of being told to go home and wait:

*“But I think it’s just the waiting*,* the waiting. Mentally*,* you’re not ready for that. You know*,* it’s kind of*,* my baby is dead. Can we just please get it out?” P5*.

#### “An awful, awful time”: devastation and subsequent pregnancies

Some women talked about the impact of their losses on subsequent pregnancies:

*“That anxiety I didn’t have before is still there after five years*,* that*,* at times of stress*,* at times of worry*,* it definitely creeps up. Especially in the subsequent pregnancy. […] It was an awful*,* awful time”. P2*.

### Theme 2: loss of baby

All participants reflected on the loss of their babies as a difficult and often heartbreaking experience. Sub-themes focused on recurrent anticipated loss, grief surrounding the loss of their baby’s future, and the pain of what felt like intangible loss.

#### ‘I knew what was coming’: anticipated loss

Women spoke about how their past experiences led to feelings of anticipated loss which intensified their distress:

*“So by the third time I was going to early pregnancy clinic when it was the same midwife who was scanning me again*,* I was convinced that just by previous experience that probably is going to be bad news.” P2*.

*“The second time I was an absolute pieces because I knew what was coming.” P1*.

#### ‘What we could have had’: grieving the loss of future

Some participants spoke about their experiences of grieving the loss of a future and what could have been:

*“It’s just sadness anda yeah*,* what we could have had.” P4*.

While one participant mentioned how a loss at later gestation had been more ‘significant’ to her than her other losses, and others felt an increase in distress between their first and second losses, none of the women distinguished between the feelings of loss and grief experienced across their miscarriages or the love they felt for lost and living children.

*“But whenever you get positive pregnancy test*,* I felt like I was having a baby and that’s what and all those miscarriages are. I still feel like that*,* you know. I have one child who thankfully is here. But there are others who aren’t*,* and they’re not*,* they’re not any less*,* you know. They are my babies.” P10*.

#### “As if it didn’t exist”: the pain of intangible loss

Some women spoke about the grief that came with the feeling that other people did not know their babies existed as ‘tangible beings’:

*“We hadn’t told anybody I was pregnant. So*,* it was just like as if it didn’t exist nearly*,* only to me and my husband. […] If your child is lost later in pregnancy or stillborn*,* people talk about their name. Not always*,* but they have a name*,* they have an identity*,* they have an existence.” P2*.

For some, even when people did know about the miscarriage, their responses to the loss caused additional pain:

*“You wouldn’t say that [‘get on with it’] to somebody that had just lost a person*,* a tangible being*,* and I think people find that hard when there hasn’t been a tangible baby or child there. They don’t really get the grief is the same.” P3*.

Likewise, for some women, these feelings of intangible loss were compounded by healthcare professional’s responses:

*“My language was very much about ‘this is my child. I am pregnant with a baby’. But their language was ‘we see nothing’. There was no mention of heartbeat. There’s no mention of baby.” P2*.

### Theme 3: isolation and guilt

#### “My fault”: guilt and RPL

Several women spoke about their fears that their losses were their fault:

*“You know*,* I drank this*,* I exerted myself too much. I must have. I didn’t*,* you know*,* wipe myself properly. My personal hygiene is not clean enough.” P8*.

Some women talked about the importance of having their baby tested as a means of alleviating their feelings of guilt:

*“I think at that stage I wanted to get a test to prove that it wasn’t my fault.” P1*.

Some women spoke about how health professionals’ comments had compounded their feelings of guilt:

*“I never actually let myself call them babies*,* or think about them in that way and*,* and it annoyed me actually when she [the midwife] tried to because I think it highlighted that I [participant emphasises the word ‘I’] had lost it.” P6*.

#### “Don’t talk about it”: isolated by miscarriage stigma

Some women talked about their difficulties in expressing their grief as much as they would like to because of the stigma surrounding miscarriage and grief:

*“And you’re always talking to your friends about your grief and you kinda think ‘Jesus are they dreading seeing me?’” P3*.

***“****Miscarriage is just so hush and taboo and people just don’t talk about it.” P2*.

#### “We were just caught adrift”: isolated by inadequate healthcare provision

Several women spoke about how the information provided to them by health professionals during their miscarriages was vague and unclear. For these women, confirmation of miscarriage was important and the delay in having that confirmation added to their feelings of isolation:

*“I think that kind wooliness of ‘there might be a miscarriage*,* but there might not be a miscarriage’*,* I don’t think that’s helpful. I think the clinician*,* as hard as it is to say*,* needs to say clearly what’s happening.” P3*.

Following confirmation of miscarriage, many women reported feeling isolated by the lack of follow-up support provided:

*“We were just caught adrift. There was no emotional support*,* there was no follow up” P3*.

Some women also mentioned the lack of support provided to their husbands:

*“He felt that nobody at any stage said to him*,* you know*,* how do you feel? Or do you need any help? So he was trying to support me through this*,* but nobody was actually helping him.” P9*.

#### ‘Did you not get a leaflet the last time?”: isolated during RPL

Some women that they did not receive the support they needed during subsequent losses, because it was assumed that their prior experiences had prepared them for another loss:

*“I think it should be explained to you like it’s the first time it’s happened to you every time*,* ‘cause it’s amazing how different each miscarriage is.” P1*.

*“I asked ‘what happens? Is there anything that I can get?’ and quote unquote [the nurse said] “Did you not get a leaflet the last time?”. And I said*,* ‘I did*,* but I didn’t keep it.’” P2*.

### Theme 4: wellbeing supports for RPL

During interviews most women talked about the things that supported their wellbeing and helped them manage their distress and navigate and make sense of their losses. Themes included the importance of compassionate healthcare, mental health support, social support, memory making and aspects of self-protection and resilience, which they developed over time.

#### “They couldn’t have done enough for you”: compassionate healthcare

A central support mentioned by participants was the sensitive and compassionate care that they received form healthcare staff, in particular midwives:

*“The midwives were amazing. Like they were so nice. So comforting. They couldn’t have done enough for you.” P5*.

Women reported how this helped them to better express and process their grief:

*“Because I got that support from her*,* I felt slightly more equipped to deal with it.” P6*.

#### “It certainly helped me”: mental health support

Some women spoke about the importance of formal and informal mental health support:

*“I had eight sessions [of CBT] and I*,* till this day*,* would still use the techniques that I was taught […] It certainly helped me.” P2*.

#### “Sometimes I think I wouldn’t be here”: social support

Some women mentioned the comfort and support provided to them by their living children and grandchildren:

*“If I hadn’t had my daughter as my safe space. I probably would have been in an even darker place because I knew you had to be there for her.” P2*.

*“But now I have a grand- daughter and without her honestly*,* sometimes I think I wouldn’t be here.” P5*.

Others reached out to their social networks for support:

*“I ended up just like thinking about people who I knew who had similar experiences and reaching out to them.” P10*.

One woman felt that the peer support she received wasn’t always helpful:

*“Peer support whenever other people are still grieving and aren’t in a healing place*,* is not good.” P2*.

#### “Physical things to remember”: objects of loss and memory making

Most of the women talked about the importance of physical objects which served as reminders of their babies:

*“I like to have physical things to remember. […] You know that they’re not just something that you forget about.” P10*.

*“I asked for pictures each time because it’s like […] it’s your first and your last picture.” P1*.

Those who did not have such objects spoke of their regret:

*“I would love to have had something to go along with my two daughters birth certificates*,* that there was something about their two siblings*,* a piece of paper to acknowledge*,* you know.” P2*.

#### “Wouldn’t let myself believe” and “You have to make your peace with it”: coping strategies and resilience following RPL

Some women talked about coping strategies that they employed following previous losses. This included maintaining an emotional detachment from baby until they were certain the pregnancy was viable:

*“We had no cot*,* we had no clothes*,* we had no anything in the house. I just wouldn’t let myself believe that it was all gonna work out.” P6*.

Some women talked about the strategies they used to help themselves build resilience during their experiences. These included availing of support that was offered to them:

*“And then in my subsequent pregnancy and they told me I could come down at any moment and be scanned and sometimes I went down twice ‘cause I was just like*,* oh*,* I’m so worried.” P1*.

Others made the decision not to risk another loss:

*“At some point you have to make your peace with it because I would have loved to have had other children and I would love for my wee girl to have a sibling*,* but you can’t*,* you know. It’s very hard to just keep chasing something that doesn’t happen because it does take a toll on you.” P10*.

## Discussion

This analysis presents a mixed-methods perspective of women’s psychological distress and wellbeing following RPL. In line with the findings of previous research [3, 7], the combined results suggest that women who experience two or more miscarriages report significant adverse impacts on psychological distress and wellbeing and experience repeated loss as devastating, isolating and guilt provoking. The paper reports findings from one of few cross-sectional studies to examine physical and experiential factors associated with distress among women who have experienced RPL. It offers a unique exploratory analysis of reported differences in distress across groups of women accounting for variations in number of miscarriages and loss at different gestation ages. Importantly, the findings also add depth to our understanding of the lived experiences of women who navigate the devastation of recurrent pregnancy loss and, in doing so, contribute to a gap in international evidence noted by others [1, 7]. These findings highlight the range of factors that can influence individual experiences of RPL and will be of value to those wishing to develop and implement targeted mental health support for these women and their partners.

### RPL and miscarriage associated distress

When compared with women who had experienced one miscarriage, women who had experienced *two or more* miscarriages demonstrated significantly more signs of distress. This finding is consistent with prior research reporting that RPL is associated with adverse psychological outcomes [3], however, differs from those of He et al. [9] who found *three or more* pregnancy losses to be associated with significantly greater depression and anxiety compared to experience of two pregnancy losses. The difference in findings may be ascribed to the use of a broader trans-diagnostic measure of distress in the current analysis in contrast to the diagnostic measures employed by He et al. [9], which may provide valuable insight into more subtle indicators of distress not yet manifested in psychopathology.

The findings of the current analysis indicate that experiences of loss, isolation, guilt and devastation increase when miscarriage happens “*again*”. Importantly, however, this does not appear to be a clear-cut relationship; those who experience a greater number of miscarriages do not always experience additive risk of miscarriage-related distress. Possible explanations for this are suggested in the qualitative analysis. For example, one potential driver, as remarked by one participant may be: “*how different each miscarriage is*” (see Theme 3), with the implication being that some miscarriages are experienced as more distressing than others. While RPL is a risk factor for adverse impact of miscarriage the heterogeneity of these experiences suggests this is not an unambiguous risk factor. Also, women in the current study spoke of how they developed resilience and employed protective coping strategies during subsequent pregnancies, which may have ameliorated their suffering and potential longer-term impacts. It worth noting that, although we did not ask survey participants if they had living children before their losses or subsequent live births, 70% of survey participants indicated they were currently living with their ‘partner and children’. Given that the reported association between childlessness and increased psychiatric morbidity following miscarriage [22], the current study may not adequately represent the potentially more devasting impacts of miscarriage on childless women. Future research should examine experiences and psychiatric outcomes among this population.

Further, while some women reported painful memories and devastating healthcare experiences, others spoke of the compassionate healthcare professionals who *“couldn’t do enough”* and supported them through their losses. These findings suggest that a broad approach to supporting those who experience RPL involving two or more losses may be warranted. This is in line with recommendations that RPL need not be defined by the number or sequencing of losses [23], and that RPL may exhibit a more nuanced association with psychological wellbeing through difference in experiences and appraisal of these.

### RPL and physical variables

Quantitative assessment of additional factors associated with miscarriage suggested that women who experienced miscarriage at a later gestation reported significantly greater psychological impacts. This is in line with previous studies which suggest that later-term miscarriage is associated with greater stress [24]. The relationship between gestational age and psychological distress is, however, not clear cut. Empirical evidence has reported conflicting relationships between gestational age at time of miscarriage and maternal wellbeing outcomes [22]. There is, hence, a need to consider individual appraisal of pregnancy loss experiences. The qualitative findings presented in this study suggest that while gestational age at loss may result in more severe feelings of loss for some, for most women the loss of a baby at any gestation can be a devastating, isolating and guilt provoking experience requiring dedicated support. Previous studies have reported the experience of miscarriage and disenfranchised grief [25, 26], and while women in the current study had a strong awareness of the shifting but persistent stigma surrounding grief and early pregnancy loss, the pain enforced by silence was evident in their narratives. Most women spoke about their continued attachment to their babies, regardless of gestational age at loss, and the importance of speaking of them as *babies* rather than *products of conception* or similar. Many spoke about the importance of retaining physical objects and memory-making activities, which seemed to play a part in making a disenfranchised grief into something tangible.

The finding of non-significant associations between distress indicators and time since most recent loss and method of conception should also be considered. Previous studies have suggested that greater distress is experienced in relation to recency of miscarriage experience [5], and with the use of assisted reproductive technology [27]. It should, however, be noted that women in current sample overwhelmingly reported natural conception of most recent pregnancy ending in miscarriage and the majority had most recently experienced miscarriage 1–2 or 3–4 years prior to participation, therefore, the effect on miscarriage-related distress may have been muted in these analyses due to habituation or recall bias for those reporting more time elapsed since miscarriage experience. However, the detailed stories related in the qualitative analysis indicated that some women remembered their miscarriages and the devastating feelings accompanying it with vivid clarity. Future research may consider specific analysis of these characteristics as risk factors for adverse psychological outcomes following pregnancy loss experiences.

### RPL and subsequent pregnancies

Considered also in the current study, is the impact of distress associated with prior miscarriage experience on subsequent pregnancies. Women described their subsequent pregnancies as *‘an awful time’* during which stress was managed with detachment and worsened for some by the expectation that having *“got the leaflet the last time”*, they should be equipped to deal with recurrent loss. In addition to the potential detrimental impact of prenatal stress and emotional detachment on women and their offspring [28, 29], previous research indicates that those who experience significant mental health difficulties are at increased risk of experiencing pregnancy loss [30]. This may contribute to what could be a vicious cycle of emotional distress and pregnancy loss experiences. It is therefore suggested that effective care and screening should consider psychological distress linked to RPL experiences as a risk factor for adverse maternal emotional and clinical outcomes [31].

### RPL and partners

Evidence has demonstrated that male partners are likewise adversely effected by miscarriage experiences [32]. While similar patterns of association are found, with successful adaptation or reduction in distress overtime following pregnancy loss, it is noted that male partners report less distress relative to their female partners [33]. Nevertheless, some women in the current study reported the negative impacts that their male partners experienced. Future research should consider the potential for didactic assessment of distress to understand distress outcomes for women, their partners (both male and female), and the couple as a unit. Research should also seek to develop and implement evidence-based provision for partner support in practice.

### RPL support

In line with other research [34], the findings of the current analysis pay testament to the vital role that healthcare and social support plays in supporting women’s health and wellbeing and helping them to build resilience in the face of devastation. The implications of this care provision are clear; compassionate, patient-centred mental health support should commence when a woman experiences her first loss. The findings that indicate distinctly increased distress from two losses onwards provide a clear indication that this support should commence at the point of the first miscarriage and then increase appropriately at the point of recurrent loss. The findings also implicate the need for training for healthcare providers in dealing with the distress of early pregnancy loss, including bereavement [35]. Future research should seek to ratify these findings, and to explore the viability of acceptance and compassion-focused intervention to support women who experience miscarriage.

## Strengths & limitations

A major strength of this analysis is that it is the first mixed methods study to examine physical and experiential factors associated with RPL in a sample of women in NI, a region with sociodemographic characteristics and healthcare services similar to many parts of the UK and other high-income countries. The quantitative data used in the current investigation were limited by their cross-sectional non-random nature, however, the use of a large sample and robust analytic techniques lend confidence to the quantitative findings. As noted, we did not ask survey participants if they had subsequent pregnancies following their miscarriage and while 70% of the study sample reported living with children, we are unable to establish if these births occurred prior to or following miscarriage experiences or whether those who reported living alone were childless. This omission did not allow sub-analysis of distress and mental well-being among these sub-groups. The qualitative analysis is limited by the fact that it involved secondary analysis of data not principally focused on those who had experienced recurrent pregnancy loss. While the sample size was small, the evidence of recurrent themes across the participants represents a valuable preliminary analysis of the experiences of this population. However, it is possible that further themes would have emerged with inclusion of a bigger sample. Further research, which can probe deeper understandings of the experience and navigation of recurrent loss among larger samples is urgently needed. In spite of these limitations, the approach adopted by this investigation represents a principled approach to methodological triangulation [36]. Data collection took place during the Covid-19 pandemic, which had an impact on maternity services in Northern Ireland. Women reported they felt frustrated and scared attending hospital appointments on their own and receiving a diagnosis of miscarriage. Many women could not access hospital services due to Covid-19 restrictions and cancelled appointments. This may have affected the overall experience of women who had a miscarriage Covid-19 and have an impact on this study results. The impacts of the pandemic on the sample are reported elsewhere [37].

## Conclusions

Women may have diverse experiences of miscarriage through recurrent losses, and this has the potential to significantly impact their wellbeing. To support women’s reproductive health and rights, acknowledgement of the grief of pregnancy loss, and compassionate approaches to care during miscarriage and subsequent pregnancies are important in reducing adverse impacts on wellbeing.

## Supplementary Information

Supplementary Material 1.

Supplementary Material 2.

Supplementary Material 3.

Supplementary Material 4.

## Data Availability

Data are not made publicly available as informed consent was not obtained for this at the time of data collection. Anonymised data relating to study analyses may be made available upon reasonable requested to the corresponding author.

## Abbreviations

NI: Northern Ireland
RPL: Recurrent pregnancy loss
RIMS: Revised Impact of Miscarriage Scale

## References

[1] Quenby S, Gallos ID, Dhillon-Smith RK, Podesek M, Stephenson MD, Fisher J, et al. Miscarriage matters: the epidemiological, physical, psychological, and economic costs of early pregnancy loss. Lancet. 2021;397(10285):1658–67. 33915094 10.1016/S0140-6736(21)00682-6

[2] Dimitriadis E, Menkhorst E, Saito S, Kutteh WH, Brosens JJ. Recurrent pregnancy loss. Nat Rev Dis Primers. 2020;6(1):98. 33303732 10.1038/s41572-020-00228-z

[3] Farren J, Mitchell-Jones N, Verbakel JY, Timmerman D, Jalmbrant M, Bourne T. The psychological impact of early pregnancy loss. Hum Reprod Update. 2018;24(6):731–49. 30204882 10.1093/humupd/dmy025

[4] Mergl R, Quaatz SM, Lemke V, Allgaier AK. Prevalence of depression and depressive symptoms in women with previous miscarriages or stillbirths – a systematic review. J Psychiatr Res. 2024;169:84–96. 38006823 10.1016/j.jpsychires.2023.11.021

[5] Farren J, Jalmbrant M, Falconieri N, Mitchell-Jones N, Bobdiwala S, Al-Memar M, et al. Posttraumatic stress, anxiety and depression following miscarriage and ectopic pregnancy: a multicenter, prospective, cohort study. Am J Obstet Gynecol. 2020;222(4):367361–367322. 10.1016/j.ajog.2019.10.102 31953115

[6] Jacob L, Polly I, Kalder M, Kostev K. Prevalence of depression, anxiety, and adjustment disorders in women with spontaneous abortion in Germany – a retrospective cohort study. Psychiatry Res. 2017;258:382–6. 28865722 10.1016/j.psychres.2017.08.064

[7] Tavoli Z, Mohammadi M, Tavoli A, Moini A, Effatpanah M, Khedmat L, et al. Quality of life and psychological distress in women with recurrent miscarriage: a comparative study. Health Qual Life Outcomes. 2018;16(1):150. 30055644 10.1186/s12955-018-0982-z PMC6064101

[8] Toffol E, Koponen P, Partonen T. Miscarriage and mental health: results of two population-based studies. Psychiatry Res. 2013;205(1–2):151–8. 22985545 10.1016/j.psychres.2012.08.029

[9] He L, Wang T, Xu H, Chen C, Liu Z, Kang X, et al. Prevalence of depression and anxiety in women with recurrent pregnancy loss and the associated risk factors. Arch Gynecol Obstet. 2019;300(4):1061–6. 31485778 10.1007/s00404-019-05264-z

[10] Herbert D, Young K, Pietrusińska M, MacBeth A. The mental health impact of perinatal loss: a systematic review and meta-analysis. J Affect Disord. 2022;297:118–29. 34678403 10.1016/j.jad.2021.10.026

[11] Lee L, McKenzie-McHarg K, Horsch A. The impact of miscarriage and stillbirth on maternal–fetal relationships: an integrative review. J Reprod Infant Psychol. 2017;35(1):32–52. 29517293 10.1080/02646838.2016.1239249

[12] Gaudet C, Séjourné N, Camborieux L, Rogers R, Chabrol H. Pregnancy after perinatal loss: association of grief, anxiety and attachment. J Reprod Infant Psychol. 2010;28(3):240–51.

[13] Hawkes A, Shields RC, Quenby S, Bick D, Parsons J, Harris B. Lived experience of recurrent miscarriage: women and their partners’ experience of subsequent pregnancy and support within an NHS specialist clinic – a qualitative study. BMJ Open. 2023;13(12):e075062. 38123186 10.1136/bmjopen-2023-075062 PMC10749006

[14] Galeotti M. The emotional needs of women who experience miscarriage in hospital settings: a mixed-methods needs assessment in Northern Ireland [PhD Thesis]. [Belfast, UK]: Queen’s Univerity Belfast; 2023. Available from: https://pure.qub.ac.uk/en/persons/martina-galeotti-2/studentTheses/. Cited 14 Feb 2025.

[15] Huffman CS, Swanson K, Lynn MR. Measuring the meaning of miscarriage: revision of the impact of miscarriage scale. J Nurs Meas. 2014;22(1):29–45. 24851662 10.1891/1061-3749.22.1.29

[16] Pourhoseingholi MA, Vahedi M, Rahimzadeh M. Sample size calculation in medical studies. Gastroenterology and Hepatology from Bed to Bench. Hepatol Bed Bench. 2013;6(1):14–7. PMC4017493 24834239

[17] Department of Health Social Services and Public Safety. Bereavement guidance after the experience of a miscarriage, stillbirth or neonatal death. Belfast UK; 2015. Available from: https://www.health-ni.gov.uk/articles/bereavement-guidance-after-experience-miscarriage-stillbirth-or-neonatal-death. Cited 25 Aug 2025.

[18] Mangiafico SS. Summary and Analysis of Extension Program Evaluation in R. VERSION 1.20.04. New Brunswick, NJ: Rutgers Cooperative Extension; 2023. Available from: https://rcompanion.org/documents/RHandbookProgramEvaluation.pdf.

[19] The Jamovi Project. Jamovi. 2024. Available from: https://www.jamovi.org.

[20] Braun V, Clarke V. Using thematic analysis in psychology. Qual Res Psychol. 2006;3(2):77–101.

[21] Cohen J. A power primer. Psychol Bull. 1992;112(1):155–9. 19565683 10.1037//0033-2909.112.1.155

[22] Lok IH, Neugebauer R. Psychological morbidity following miscarriage. Best Pract Res Clin Obstet Gynaecol. 2007;21(2):229–47. 17317322 10.1016/j.bpobgyn.2006.11.007

[23] ESHRE Guideline Group on RPL, Bender Atik R, Christiansen OB, Elson J, Kolte AM, Lewis S, et al. ESHRE guideline: recurrent pregnancy loss. Hum Reprod Open. 2018;2018(2):hoy004. 31486805 10.1093/hropen/hoy004 PMC6276652

[24] Jansson C, Volgsten H, Huffman C, Skoog Svanberg A, Swanson KM, Stavreus-Evers A. Validation of the revised impact of miscarriage scale for Swedish conditions and comparison between Swedish and American couples’ experiences after miscarriage. Eur J Contracept Reprod Health Care. 2017;22(6):412–7. 29250992 10.1080/13625187.2017.1409346

[25] Cassidy PR. The disenfranchisement of perinatal grief: how silence, silencing and self-censorship complicate bereavement (a mixed methods study). Omega (Westport). 2023;88(2):709–31. 34632863 10.1177/00302228211050500

[26] Mulvihill A, Walsh T. Pregnancy loss in rural Ireland: an experience of disenfranchised grief. Br J Soc Work. 2014;44(8):2290–306.

[27] Cheung C, Chan C, Ng E. Stress and anxiety-depression levels following first‐trimester miscarriage: a comparison between women who conceived naturally and women who conceived with assisted reproduction. BJOG. 2013;120(9):1090–7. 23631687 10.1111/1471-0528.12251

[28] Bergman K, Sarkar P, Glover V, O’Connor TG. Maternal prenatal cortisol and infant cognitive development: moderation by infant–mother attachment. Biol Psychiatry. 2010;67(11):1026–32. 20188350 10.1016/j.biopsych.2010.01.002 PMC2872196

[29] Coussons-Read ME. Effects of prenatal stress on pregnancy and human development: mechanisms and pathways. Obstet Med. 2013;6(2):52–7. 27757157 10.1177/1753495X12473751 PMC5052760

[30] Magnus MC, Havdahl A, Morken NH, Wensaas KA, Wilcox AJ, Håberg SE. Risk of miscarriage in women with psychiatric disorders. Br J Psychiatry. 2021;219(3):501–6. 33448259 10.1192/bjp.2020.259 PMC7611718

[31] Riddle JN, Hopkins T, Yeaton-Massey A, Hellberg S. No baby to bring home: perinatal loss, infertility, and mental illness—overview and recommendations for care. Curr Psychiatry Rep. 2023;25(11):747–57. 37878138 10.1007/s11920-023-01469-x

[32] Voss P, Schick M, Langer L, Ainsworth A, Ditzen B, Strowitzki T, et al. Recurrent pregnancy loss: a shared stressor—couple-orientated psychological research findings. Fertil Steril. 2020;114(6):1288–96. 33039130 10.1016/j.fertnstert.2020.08.1421

[33] Farren J, Jalmbrant M, Falconieri N, Mitchell-Jones N, Bobdiwala S, Al‐Memar M, et al. Differences in post‐traumatic stress, anxiety and depression following miscarriage or ectopic pregnancy between women and their partners: multicenter prospective cohort study. Ultrasound Obstet Gynecol. 2021;57(1):141–8. 33032364 10.1002/uog.23147

[34] Galeotti M, Mitchell G, Tomlinson M, Aventin A. Factors affecting the emotional wellbeing of women and men who experience miscarriage in hospital settings: a scoping review. BMC Pregnancy Childbirth. 2022;22(1):270. 35361132 10.1186/s12884-022-04585-3 PMC8974061

[35] Galeotti M, Heaney S, Robinson M, Aventin Á. Evaluation of a pregnancy loss education intervention for undergraduate nursing students in Northern Ireland: a pre- and post-test study. BMC Nurs. 2023;22(1):268. 37580730 10.1186/s12912-023-01408-4 PMC10424365

[36] Braun V, Clarke V, Boulton E, Davey L, McEvoy C. The online survey as a qualitative research tool. Int J Soc Res Methodol. 2021;24(6):641–54.

[37] Heaney S, Galeotti M, Aventin Á. Pregnancy loss following miscarriage and termination of pregnancy for medical reasons during the COVID-19 pandemic: a thematic analysis of women’s experiences of healthcare on the Island of Ireland. BMC Pregnancy Childbirth. 2023;23(1):529. 37480006 10.1186/s12884-023-05839-4 PMC10360341

