# Supplementary material for: Recurrent pregnancy loss, psychological distress and wellbeing support for women: a mixed-methods analysis
Source: BMC Womens Health. 2025 Nov 3;25:535. doi: 10.1186/s12905-025-04079-2 (PMC12581269; doi:10.1186/s12905-025-04079-2)
Supplement: Supplementary file 1 — Supplementary Material 1. [file 12905_2025_4079_MOESM1_ESM.pdf]

The emotional needs of women who experience miscarriage in hospital settings: a mixed-methods needs assessment in Northern Ireland (Galeotti, 2023)

Online Survey Questions

Section	Questions	Reference
**1. History of miscarriage**	1. How many times have you had a pregnancy which resulted in a miscarriage?<br>2. When did you experience your miscarriage?<br>3. How many weeks pregnant were you when you had your miscarriage?<br>4. When you suspected you were miscarrying, which care option did you access first?<br>5. Were you re-directed to another service?<br>6. If, yes where? (free text)	1. Questions created by research team<br>2. Questions created by research team<br>3. Questions created by research team<br>4. Question adapted from (AIMS Ireland 2009)<br>5. Questions created by research team<br>6. Questions created by research team
**2. Impact of miscarriage**	1. Did you experience any mental health issues following your miscarriage (e.g. depression, anxiety and post-traumatic stress disorder)?<br>2. Did you receive a diagnosis of any mental health disorders (e.g. depression, anxiety and post-traumatic stress disorder) following your miscarriage?<br>3. Reverse Impact Miscarriage Scale (16 Items)	1. Questions created by research team<br>2. Questions created by research team<br>3. (Huffman, Swanson, and Lynn 2014, Jansson et al. 2017)
**3. Interaction with health professionals**	1. The news that I was having a miscarriage was clearly explained to me by health professionals<br>2. The news I was having a miscarriage was communicated sensitively to me by health professionals<br>3. The news I was experiencing a miscarriage was communicated to me in an environment where I could have privacy<br>4. I was kept updated about my plan of care by health professionals	1. Questions created by research team<br>2. Question adapted from (AIMS Ireland 2009)<br>3. Questions created by research team<br>4. Questions created by research team<br>5. Questions adapted from (AIMS Ireland 2009)

	5. My feelings about my miscarriage were acknowledged appropriately by health professionals<br>6. I was able to express my feelings to health professionals<br>7. I was able to express my worries and fears to health professionals<br>8. My feelings were taken into account by health professionals<br>9. My religious or spiritual beliefs were taken into account by health professionals<br>10. During my hospital visit a health professional explained to me the possible physical implications of experiencing miscarriage (e.g. pain and bleeding)<br>11. During my hospital visit a health professional explained to me the possible emotional implications of experiencing miscarriage (e.g. grief, anxiety, depression, trauma)	6. Questions created by research team<br>7. Questions created by research team<br>8. Questions created by research team<br>9. Questions created by research team<br>10. Questions created by research team<br>11. Questions created by research team
**4. Management of miscarriage**	1. What treatment did you have during your miscarriage?<br>2. Were you given the opportunity to choose the kind of treatment you would have?<br><br>**Section 4a.**<br><br>1. Why did you have this particular treatment? (Tick as many boxes as you like)<br>2. During my hospital visit, all the treatment options (expectant, medical and surgical management) were clearly explained to me by health professionals<br>3. My opinion was taken into account by health professionals while deciding on treatment options for miscarriage<br>4. I was given enough information about treatment options by health professionals	1. Questions created by research team<br>2. Questions created by research team<br><br>1. Questions created by research team<br>2. Questions created by research team<br>3. Questions created by research team<br>4. Questions created by research team<br>5. Questions created by research team<br>5. Questions created by research team<br>6. Questions created by research team

	5. I was given enough time to think about treatment options before making my decision<br>6. My feelings were taken into account by health professionals while discussing treatment options for miscarriage<br>7. My worries and fears were taken into account by health professionals while discussing treatment options<br>8. It was important to me to discuss my treatment options with health professionals<br>9. I felt empowered to be able to discuss my treatment options with health professionals<br><br>**Section 4b**<br><br>1. Why were you not able to choose your treatment? (select one of the following statements)<br>2. During my hospital visit, my treatment options (expectant, medical and surgical management) were clearly explained to me by health professionals<br>3. I had enough time to discuss my treatment with health professionals<br>4. My worries and fears were addressed by health professionals while discussing my treatment<br>5. I was upset or angry that I could not choose my treatment<br>6. I understood why I was not able to choose my treatment	7. Questions created by research team<br>8. Questions created by research team<br>9. Questions created by research team<br><br>1. Questions created by research team<br>2. Questions created by research team<br>3. Questions created by research team<br>4. Questions created by research team<br>5. Questions created by research team<br>6. Questions created by research team
**5. Miscarriage in different settings**	1. Were you discharged home to have your miscarriage?<br><br>**5a. Miscarriage during hospital admission**<br>1. Which of the following people helped you feel emotionally supported during your hospital stay? (Tick as many boxes as you like)<br>2. Which of the following things helped	1. Questions created by research team<br><br>1. Question adapted from (AIMS Ireland 2009)<br>2. Questions created by research team

	you feel emotionally supported during your hospital stay? (Tick as many boxes as you like)<br>3. I felt relieved that I was able to have my miscarriage in hospital<br>4. I felt comfortable knowing I was able to have my miscarriage in hospital<br>5. I felt lonely while having my miscarriage in hospital<br>6. I felt isolated while having my miscarriage in hospital<br><br>**5b. Miscarriage at home**<br><br>1. I had enough information when leaving hospital to feel prepared to have my miscarriage at home<br>2. Which of the following people helped you feel emotionally supported while having your miscarriage at home? (Tick as many boxes as you like)<br>3. Which of the following things helped you feel emotionally supported while having your miscarriage at home? (Tick as many boxes as you like)<br>4. I felt lonely while having my miscarriage at home<br>5. I felt isolated while having my miscarriage at home<br>6. I felt relieved that I was able to have my miscarriage at home<br>7. I felt more comfortable knowing I was able to have my miscarriage at home.	3. Questions created by research team<br>4. Questions created by research team<br>5. Questions created by research team<br>6. Questions created by research team<br><br>1. Questions created by research team<br>2. Question adapted from (AIMS Ireland 2009)<br>3. Questions created by research team<br>4. Questions created by research team<br>5. Questions created by research team<br>6. Questions created by research team<br>7. Questions created by research team
**7. Follow-up care**	1. Before leaving the hospital, I was provided with contact numbers (e.g. community services, Early Pregnancy Units) in case I needed further assistance<br>2. I was advised to make an appointment to receive follow up care with (named different HP)<br>3. Following my miscarriage, I was offered information on emotional or	1. Questions created by research team<br>2. Question adapted from (AIMS Ireland 2009)<br>3. Questions created by research team<br>4. Questions created by research team

	mental health support services (e.g. counselling, bereavement care, support groups)<br>4. Who offered you this information? (Tick as many boxes as you like)	
5. **Emotional support**	1. After your miscarriage, did you access any kind of supportive services?<br>2. Why did you not access any additional supportive services support services? (Tick as many boxes as you like)<br>3. Which services did you use? (Tick as many boxes as you like)<br>4. Looking back, what helped you emotionally, after your miscarriage? (Tick as many boxes as you like)<br>5. Please indicate your level of satisfaction with the following statements:<br>6. The emotional support I received from hospital staff at the time of miscarriage<br>7. The psychological/emotional follow-up services after my hospital visit<br>8. The emotional support I received in hospital could be improved	1. Question adapted from (AIMS Ireland 2009)<br>2. Questions created by research team<br>3. Questions created by research team<br>4. Questions created by research team<br>6. Question adapted from (AIMS Ireland 2009)<br>7. Questions created by research team<br>8. Question adapted from (AIMS Ireland 2009)<br>9. Question adapted from (AIMS Ireland 2009)
6. **About you**	1. Please indicate your age range<br>2. What is your current marital status?<br>3. What is the highest level of education you completed?<br>4. What is your main employment status?	
7. **Open-ended questions**	1. Do you have any suggestions about how your emotional needs might have been better supported in hospital settings, while you were experiencing your miscarriage?<br>2. How did Covid-19 had impacted on your hospital miscarriage in hospital settings?	1. Question adapted from (AIMS Ireland 2009)<br>2. Questions created by research team
