# Supplementary material for: Recurrent pregnancy loss, psychological distress and wellbeing support for women: a mixed-methods analysis
Source: BMC Womens Health. 2025 Nov 3;25:535. doi: 10.1186/s12905-025-04079-2 (PMC12581269; doi:10.1186/s12905-025-04079-2)
Supplement: Supplementary file 2 — Supplementary Material 2. [file 12905_2025_4079_MOESM2_ESM.pdf]

Results

Correlation Matrix

Pregnancy Loss Variables Correlated (Spearman's) with Impact of Miscarriage Scale Outcomes.

Correlation Matrix

		NPreg	MisscTime	WkPreg	RIMS_total	RIMss_Devastation	RIMss_Loss	RIMss_Isolation
NPreg	Spearman's rho	—						
	df	—						
	p-value	—						
MisscTime	Spearman's rho	0.0677	—					
	df	837	—					
	p-value	0.050	—					
WkPreg	Spearman's rho	0.1272	0.0149	—				
	df	837	837	—				
	p-value	<.001	0.666	—				
RIMS_total	Spearman's rho	0.1889	-0.0130	0.1341	—			
	df	837	837	837	—			
	p-value	<.001	0.707	<.001	—			
RIMss_Devastation	Spearman's rho	0.2012	-0.0422	0.1188	0.7959	—		
	df	837	837	837	837	—		
	p-value	<.001	0.222	<.001	<.001	—		
RIMss_Loss	Spearman's rho	0.1574	-0.0029	0.1898	0.8230	0.6222	—	
	df	837	837	837	837	837	—	
	p-value	<.001	0.932	<.001	<.001	<.001	—	
RIMss_Isolation	Spearman's rho	0.1647	0.0052	0.0888	0.9057	0.5800	0.5934	—
	df	837	837	837	837	837	837	—
	p-value	<.001	0.880	0.010	<.001	<.001	<.001	—

Scale Reliability Analysis

Internal Reliability (Cronbach's α) for RIMS.

Scale Reliability Statistics

	Cronbach's α
scale	0.9076

Descriptives

Detailed Sample Demography. Frequencies and effective percentages (pct of available data per item) reported.

Descriptives

	NPreg	MisscTime	WkPreg	AgeCat	Educ	MarStat	Concieve_Method
N	839	839	839	723	723	722	839
Missing	0	0	0	116	116	117	0
Mean	1.709	2.341	3.133	2.788	4.564		
Median	1	2	3	3	5		
Standard deviation	0.9898	0.9979	0.8595	0.7578	1.248		
Minimum	1	1	1	1	1		
Maximum	4	4	7	4	6		

Frequencies

Frequencies of NPreg

NPreg	Counts	% of Total	Cumulative %
1	489	58.28%	58.28%
2	184	21.93%	80.21%
3	87	10.37%	90.58%
4+	79	9.42%	100.00%

Frequencies of MisscTime

MisscTime	Counts	% of Total	Cumulative %
Less than a year ago	199	23.72%	23.72%
1-2 years ago	279	33.25%	56.97%
3-4 year ago	237	28.25%	85.22%
5 years ago	124	14.78%	100.00%

Frequencies of WkPreg

WkPreg	Counts	% of Total	Cumulative %
Less than 4 weeks	6	0.72%	0.72%
Between 4 and 6 weeks	113	13.47%	14.18%
Between 7 and 12 weeks	583	69.49%	83.67%
Between 13 and 16 weeks	65	7.75%	91.42%
Between 17 and 20 weeks	46	5.48%	96.90%
Between 21 and 24 weeks	24	2.86%	99.76%
Don’t know	2	0.24%	100.00%

Frequencies of AgeCat

AgeCat	Counts	% of Total	Cumulative %
16-25	48	6.64%	6.64%
26-30	156	21.58%	28.22%
31-39	420	58.09%	86.31%
40-49	99	13.69%	100.00%

Frequencies of MarStat

MarStat	Counts	% of Total	Cumulative %
Alone	23	3.19%	3.19%
With partner	191	26.45%	29.64%
With partner and other children	507	70.22%	99.86%
With partner and other people	1	0.14%	100.00%

Frequencies of Concieve_Method

Concieve_Method	Counts	% of Total	Cumulative %
Natural conception	800	95.35%	95.35%
Assisted reproductive therapies	39	4.65%	100.00%

Frequencies of Educ

Educ	Counts	% of Total	Cumulative %
No formal qualification	3	0.41%	0.41%
Standard grade/GCSE	64	8.85%	9.27%
Higher/A-Levels	93	12.86%	22.13%
College	95	13.14%	35.27%
BSc Degree	298	41.22%	76.49%
Higher degree	170	23.51%	100.00%

Descriptives

Descriptive statistics for outcome measure(s). Dependent variables were found to violate assumptions of normality (All Shapiro-Wilk $p < .001$), therefore non-parametric inferential statistics are used.

Descriptives

	RIMss_Devastation	RIMss_Loss	RIMss_Isolation	RIMS_total
N	839	839	839	839
Mean	17.68	17.29	18.68	53.65
Median	19.00	18.00	19.00	56.00
Standard deviation	2.705	2.968	4.235	8.619
IQR	4.000	4.000	6.000	11.00
Minimum	6.000	5.000	6.000	17.00
Maximum	20.00	20.00	24.00	64.00
Skewness	-1.501	-1.279	-0.7639	-1.110
Std. error skewness	0.08442	0.08442	0.08442	0.08442
Kurtosis	2.416	1.328	-0.06374	1.081
Std. error kurtosis	0.1686	0.1686	0.1686	0.1686
Shapiro-Wilk W	0.8149	0.8428	0.9304	0.9076
Shapiro-Wilk p	<.001	<.001	<.001	<.001
25th percentile	16.00	16.00	16.00	49.00
50th percentile	19.00	18.00	19.00	56.00
75th percentile	20.00	20.00	22.00	60.00

Plots

RIMss_Devastation

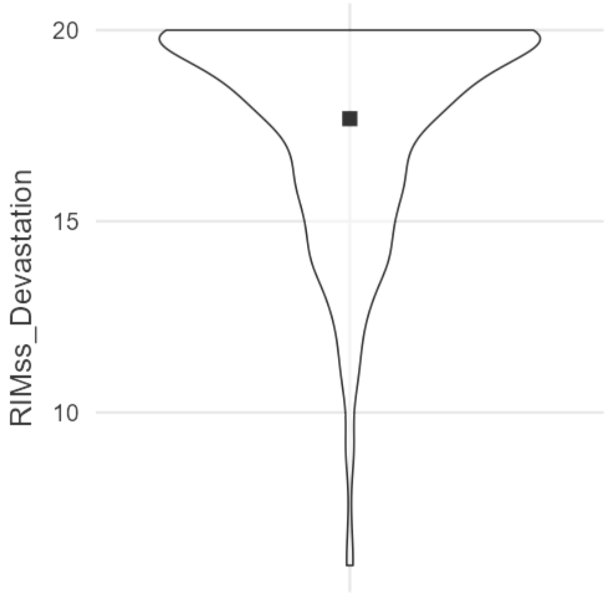

RIMss_Loss

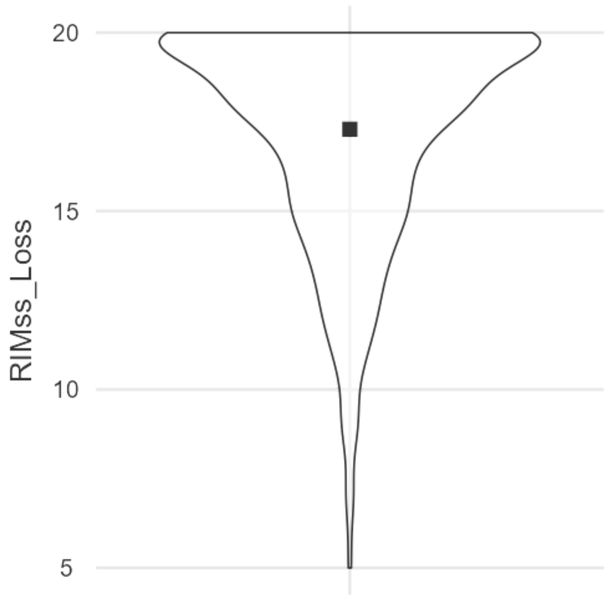

RIMss_Isolation

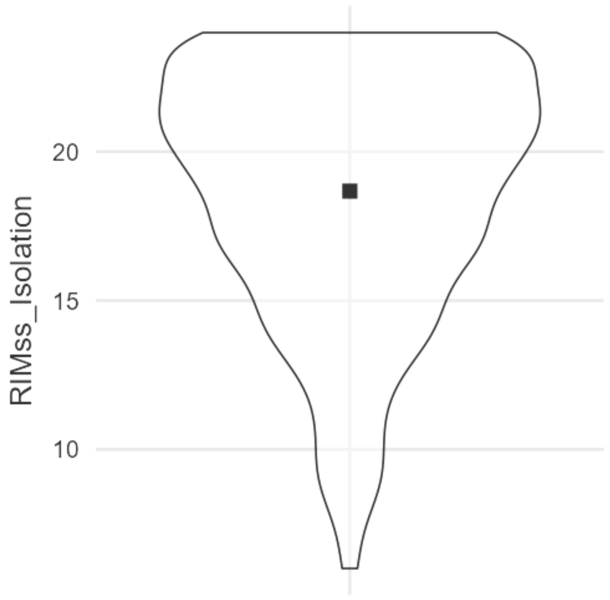

RIMS_total

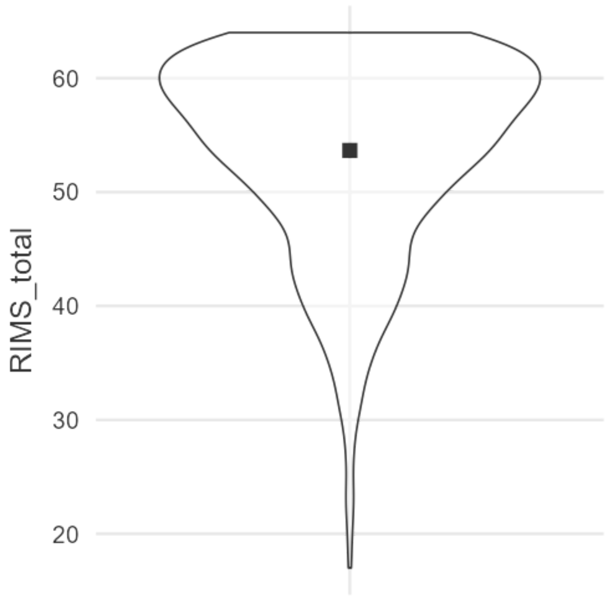

One-Way ANOVA (Non-parametric)

Test of association between number of miscarriage experiences and outcome measures.

Kruskal-Wallis

	χ^2	df	p	ε^2
RIMss_Devastation	34.61	3	<.001	0.04129
RIMss_Loss	21.29	3	<.001	0.02541
RIMss_Isolation	22.79	3	<.001	0.02720
RIMS_total	30.14	3	<.001	0.03597

Dwass-Steel-Critchlow-Fligner pairwise comparisons

Pairwise comparisons - RIMss_Devastation

		W	p
1	2	5.5752	<.001
1	3	5.9558	<.001
1	4+	5.1593	0.002
2	3	1.5305	0.700
2	4+	1.2393	0.817
3	4+	-0.1492	1.000

Pairwise comparisons - RIMss_Loss

		W	p
1	2	4.4326	0.009
1	3	4.6457	0.006
1	4+	4.0046	0.024
2	3	1.3733	0.766
2	4+	0.8344	0.935
3	4+	-0.3620	0.994

Pairwise comparisons - RIMss_Isolation

		W	p
1	2	4.3726	0.011
1	3	4.2854	0.013
1	4+	4.9428	0.003
2	3	0.6290	0.971
2	4+	1.5739	0.682
3	4+	0.9202	0.916

Pairwise comparisons - RIMS_total

		W	p
1	2	5.2573	0.001
1	3	5.2273	0.001
1	4+	5.2233	0.001
2	3	0.9631	0.905
2	4+	1.2965	0.796
3	4+	0.3964	0.992

One-Way ANOVA (Non-parametric)

Test of association between gestational age of most recent miscarriage and outcome measures.

Kruskal-Wallis

	χ^2	df	p	ε^2
RIMss_Devastation	17.58	6	0.007	0.02098
RIMss_Loss	47.23	6	<.001	0.05636
RIMss_Isolation	12.17	6	0.058	0.01453
RIMS_total	23.27	6	<.001	0.02777

Dwass-Steel-Critchlow-Fligner pairwise comparisons

Pairwise comparisons - RIMss_Devastation

		W	p
Less than 4 weeks	Between 4 and 6 weeks	-0.08742	1.000
Less than 4 weeks	Between 7 and 12 weeks	0.25265	1.000
Less than 4 weeks	Between 13 and 16 weeks	0.75317	0.998
Less than 4 weeks	Between 17 and 20 weeks	2.05569	0.772
Less than 4 weeks	Between 21 and 24 weeks	0.95526	0.994
Less than 4 weeks	Don't know	2.44600	0.596
Between 4 and 6 weeks	Between 7 and 12 weeks	1.32498	0.967
Between 4 and 6 weeks	Between 13 and 16 weeks	2.30097	0.665
Between 4 and 6 weeks	Between 17 and 20 weeks	4.54354	0.022
Between 4 and 6 weeks	Between 21 and 24 weeks	2.62310	0.511
Between 4 and 6 weeks	Don't know	2.46845	0.586
Between 7 and 12 weeks	Between 13 and 16 weeks	1.73403	0.884
Between 7 and 12 weeks	Between 17 and 20 weeks	4.45253	0.027
Between 7 and 12 weeks	Between 21 and 24 weeks	2.16976	0.724
Between 7 and 12 weeks	Don't know	2.41156	0.613
Between 13 and 16 weeks	Between 17 and 20 weeks	2.52221	0.560
Between 13 and 16 weeks	Between 21 and 24 weeks	0.97682	0.993
Between 13 and 16 weeks	Don't know	2.21522	0.704
Between 17 and 20 weeks	Between 21 and 24 weeks	-0.99538	0.992
Between 17 and 20 weeks	Don't know	1.57254	0.925
Between 21 and 24 weeks	Don't know	1.90029	0.832

Pairwise comparisons - RIMss_Loss

		W	p
Less than 4 weeks	Between 4 and 6 weeks	0.3131	1.000
Less than 4 weeks	Between 7 and 12 weeks	0.7471	0.998
Less than 4 weeks	Between 13 and 16 weeks	1.8899	0.835
Less than 4 weeks	Between 17 and 20 weeks	4.3405	0.035
Less than 4 weeks	Between 21 and 24 weeks	3.6243	0.138
Less than 4 weeks	Don't know	-0.4892	1.000
Between 4 and 6 weeks	Between 7 and 12 weeks	1.3561	0.963
Between 4 and 6 weeks	Between 13 and 16 weeks	3.3002	0.228
Between 4 and 6 weeks	Between 17 and 20 weeks	7.0841	<.001
Between 4 and 6 weeks	Between 21 and 24 weeks	5.2001	0.004
Between 4 and 6 weeks	Don't know	-0.1686	1.000
Between 7 and 12 weeks	Between 13 and 16 weeks	3.0182	0.333
Between 7 and 12 weeks	Between 17 and 20 weeks	7.5636	<.001
Between 7 and 12 weeks	Between 21 and 24 weeks	5.2970	0.003
Between 7 and 12 weeks	Don't know	-0.3968	1.000
Between 13 and 16 weeks	Between 17 and 20 weeks	4.4906	0.025
Between 13 and 16 weeks	Between 21 and 24 weeks	3.3269	0.219
Between 13 and 16 weeks	Don't know	-1.2864	0.971
Between 17 and 20 weeks	Between 21 and 24 weeks	-0.2660	1.000
Between 17 and 20 weeks	Don't know	-2.7338	0.458
Between 21 and 24 weeks	Don't know	-2.6586	0.494

Pairwise comparisons - RIMss_Isolation

		W	p
Less than 4 weeks	Between 4 and 6 weeks	-0.1121	1.000
Less than 4 weeks	Between 7 and 12 weeks	0.3029	1.000
Less than 4 weeks	Between 13 and 16 weeks	0.5440	1.000
Less than 4 weeks	Between 17 and 20 weeks	1.8212	0.858
Less than 4 weeks	Between 21 and 24 weeks	0.6640	0.999
Less than 4 weeks	Don’t know	-1.6699	0.902
Between 4 and 6 weeks	Between 7 and 12 weeks	1.6423	0.909
Between 4 and 6 weeks	Between 13 and 16 weeks	1.8519	0.848
Between 4 and 6 weeks	Between 17 and 20 weeks	4.2581	0.042
Between 4 and 6 weeks	Between 21 and 24 weeks	1.5311	0.933
Between 4 and 6 weeks	Don’t know	-1.4881	0.942
Between 7 and 12 weeks	Between 13 and 16 weeks	1.1174	0.986
Between 7 and 12 weeks	Between 17 and 20 weeks	3.9094	0.083
Between 7 and 12 weeks	Between 21 and 24 weeks	0.8715	0.996
Between 7 and 12 weeks	Don’t know	-1.8378	0.853
Between 13 and 16 weeks	Between 17 and 20 weeks	2.1595	0.729
Between 13 and 16 weeks	Between 21 and 24 weeks	0.1184	1.000
Between 13 and 16 weeks	Don’t know	-1.8620	0.845
Between 17 and 20 weeks	Between 21 and 24 weeks	-1.6090	0.917
Between 17 and 20 weeks	Don’t know	-2.4731	0.583
Between 21 and 24 weeks	Don’t know	-1.9167	0.826

Pairwise comparisons - RIMS_total

		W	p
Less than 4 weeks	Between 4 and 6 weeks	-0.03439	1.000
Less than 4 weeks	Between 7 and 12 weeks	0.28844	1.000
Less than 4 weeks	Between 13 and 16 weeks	1.02571	0.991
Less than 4 weeks	Between 17 and 20 weeks	2.22504	0.700
Less than 4 weeks	Between 21 and 24 weeks	1.54791	0.930
Less than 4 weeks	Don't know	-0.71568	0.999
Between 4 and 6 weeks	Between 7 and 12 weeks	1.39705	0.957
Between 4 and 6 weeks	Between 13 and 16 weeks	2.49871	0.571
Between 4 and 6 weeks	Between 17 and 20 weeks	5.45009	0.002
Between 4 and 6 weeks	Between 21 and 24 weeks	3.03514	0.326
Between 4 and 6 weeks	Don't know	-0.31806	1.000
Between 7 and 12 weeks	Between 13 and 16 weeks	2.18500	0.718
Between 7 and 12 weeks	Between 17 and 20 weeks	5.70503	0.001
Between 7 and 12 weeks	Between 21 and 24 weeks	2.70282	0.473
Between 7 and 12 weeks	Don't know	-0.76827	0.998
Between 13 and 16 weeks	Between 17 and 20 weeks	3.12043	0.292
Between 13 and 16 weeks	Between 21 and 24 weeks	1.04176	0.990
Between 13 and 16 weeks	Don't know	-1.48874	0.942
Between 17 and 20 weeks	Between 21 and 24 weeks	-1.25143	0.975
Between 17 and 20 weeks	Don't know	-2.31810	0.657

Pairwise comparisons - RIMS_total

		W	p
Between 21 and 24 weeks	Don’t know	-1.70953	0.891
