# Supplementary material for: Recurrent pregnancy loss, psychological distress and wellbeing support for women: a mixed-methods analysis
Source: BMC Womens Health. 2025 Nov 3;25:535. doi: 10.1186/s12905-025-04079-2 (PMC12581269; doi:10.1186/s12905-025-04079-2)
Supplement: Supplementary file 3 — Supplementary Material 3. [file 12905_2025_4079_MOESM3_ESM.pdf]

The emotional needs of women who experience miscarriage in hospital settings: a mixed-methods needs assessment in Northern Ireland (Galeotti, 2023)

Semi-structured Interview Topic Guide for women

Experience of miscarriage

1. *Can you tell me about your experience of miscarriage?*

Prompts:

- Number of pregnancies ending with a miscarriage
- When miscarriage/ miscarriages occurred
- Emotions felt with when the miscarriage occurred

If women experienced more than one miscarriage: thinking about your most recent experience of miscarriage

2. *Could you talk me through what happened when you discovered something was wrong?*

Prompts:

- Hospital department attended (i.e. Emergency Department, GP surgery, Early Pregnancy Assessment Units
- Re-direction to another department
- Waiting time

3. *Can you tell me about your experiences the first time you encountered hospital staff?*

4. *Can you tell me what happened after your first encounter with staff?*

Prompts:

- Waiting time
- Did you know where to go
- Did you suspect miscarriage

Provision of information

5. *Can you tell me about your experience of when you received the news something was wrong with your pregnancy?*

Prompts:

- Information received: where they enough, comprehensive, used lay terms?
- Information on physical and mental implication of miscarriage
- Satisfaction with emotional support while receiving the news

- Emotional support needed by women at this time

6. *How do you feel about the way health professionals spoke to you?*

Prompts

- *Example of positive interaction*
- *Example of negative interaction*
- *How did they feel*

Interaction with health professionals

7. *What healthcare professionals did you come into contact with during your time in hospital and what role did they play?*

Prompts:

- Emotional support received by health professionals
- Were address worries and needs addressed
- Examples when worries and needs were addressed

Management of miscarriage

8. *Can you tell me about your experience of the treatment of miscarriage you received?*

Prompts:

- Were you able to choose your treatment? Why?
- Information received about treatment/treatment options/were they equally explained
- Enough information about treatment option
- Was it an informed choice?
- How did they feel about the treatment received? Why did they choose a treatment?
- How did they feel about not choosing a treatment? How this effected their emotional wellbeing?
- Miscarry at home/hospital

9. *Can you tell me about your experience of miscarrying at home/ hospital?*

Prompts:

- Emotional support received
- How did they feel? Lonely/relief
- Were they emotionally supported?

**Follow-up care**
<u>Can you tell me about your experience with follow-up care after leaving the hospital?</u><br><br>*Prompts:*<br>• Support received while leaving the hospital (medical advice, medical treatment, psychological support)<br>• Were they directed to emotional/psychological support? Did they use it? Why? Did you use any extra hospital emotional/psychological support? i.e. Family and friends, charities, private counselling<br>• Did follow up support prepared for the life after miscarriage? Future pregnancy /Explaining miscarriage to young children/Taking time off work/Sexuality/Intimacy/ Spirituality
**Rounding off**
1. What might have improved your overall experience of care?<br>**2.** How do you feel Covid-19 pandemic has impacted your experience of miscarriage in hospital? (**Question to ask only to women who experienced miscarriage during Covid-19 pandemic)**<br>3. Is there anything you would like to add that I did not ask you?<br><br>4. Do you have any questions about this project?<br>5. What would you like to see come out of it?
