# Supplementary material for: Recurrent pregnancy loss, psychological distress and wellbeing support for women: a mixed-methods analysis
Source: BMC Womens Health. 2025 Nov 3;25:535. doi: 10.1186/s12905-025-04079-2 (PMC12581269; doi:10.1186/s12905-025-04079-2)
Supplement: Supplementary file 4 — Supplementary Material 4. [file 12905_2025_4079_MOESM4_ESM.pdf]

Recurrent Pregnancy Loss, Distress, and Wellbeing Support: A Mixed-Methods Study of Women in Northern Ireland

Supplementary File 3: Illustrative quotes identified by thematic analysis of interview data

Themes and Sub-themes	Illustrative participant quotes
**1: Devastating Event**	
1A: "Over and over and over": The devastating impact of RPL	*"Normally I make it to the car before I start crying. And that time I, I sat at the end of the corridor and cried." P1*<br><br>*"[I] was sort of in a state of shock. [...] didn't know what was going on." P7*<br><br>*"And this happens over and over and over. Every pregnancy, every miscarriage, and even when you are pregnant. You're on tenterhooks, your, your, your, your nerves is away." P8*<br><br>*"I developed severe anxiety [...] I didn't want to leave the house. I was crying all the time. [...] It has to be that [the miscarriages] because I never had mental health problems before." P2*<br><br>*" [I was on a] plane in the middle of the night and I was watching a movie and there is a gunshot injury and a massive thoracic trauma and a lot of bleeding and I had a full blown panic attack on the plane." P6*<br><br>*"I don't feel well mentally because in your head you're going over the pictures of everything that the scenario, the scenes, and everything that's happened to you ever since in your head [...] over and over again." P8*<br><br>*"I want to highlight that it's not just a physical thing. There is an emotional impact on people and it's much more long lasting than what the physical impact is." P10*<br><br>*"[My husband] was very upset, you know, he was, he was crying. It was the first time I've ever seen him cry." P9*<br><br>*"My husband was on a great job, walked out and didn't go back. Just went off sick and then then decided to hand his notice in and that was him. So it had a big impact on my husband as well, you know?" P5*
1B: "Full-on-labour" and "The Same Room": Devastating memories	*"I had a full-on labour, very intense physical experience which I was completely unprepared for (participant starts to cry)." P4*<br><br>*"I bled really heavily at home and lost 4 litres of blood. I can remember lying on my bed flat and blood was collecting up right in the back of my hair and then like rushing to the toilet and [..] for two days afterwards there were splashes of blood on the ceiling." P6*

	"It's a fetus, it's the size of your hand, it's like a tiny little dot. What are you meant to do whenever you go home and gave birth to that in your bathroom? [...] the excessive bleeding and having your baby that experience is quite traumatic, like, I can't get that vision out of my head." P8<br><br>*[The midwife said], 'Aww, have you lost it? That's awful. Sure, you can try again.' I was alone in a waiting room." P2*<br><br>*"But they said to me 'you're not gonna get a commode. You can go to the toilet and there's not enough staff.' I said "OK, alright, fair enough." So I needed to use the bathroom, got to the toilet, started to bleed all over the place, giant clots, and collapsed on the floor." P8*<br><br>*"The miscarriage itself was devastating but the way that I was spoken to about it, I think it was nearly worse. It was like the child I had didn't matter." P3*<br><br>*"I was like, I can't believe I have to go to that 'miscarriage room' for my first scan. [...] Then they told me there was no heartbeat, that was quite upsetting, I was already stressed about going to that room and then it was like it happened again." P1*<br><br>*"The hard thing was we had to go back to the same room [where we had our previous miscarriage]." P5*<br><br>*"I've had a lot of scans obviously because of my circumstances [...] but you are brought back into the same room over and over again so you're like re-living what happened the first time, you know it was quite difficult." P10*
1C: "Mentally you're not ready for that": A devastating wait	*"I waited a good 7 hours. And the worst thing was, the worst few hours of that was because my cervix was open, and I was so scared that the baby was gonna fall out." P1*<br><br>*"...the bathroom floor was completely flooded. [...] so I can remember coming out holding my trousers up shouting down the hall and one of the janitors came down to help me, got wheelchair and took me up to gynae, but I had to wait a good few hours 'cause they said there was no doctor or anybody available to see me" P8*<br><br>*"That was the more annoying, heart-breaking thing that we were just told, 'just go home and wait'. And nobody can go home and just wait, you know?" P5*<br><br>*"But I think it's just the waiting, the waiting. Mentally, you're not ready for that. You know, it's kind of, my baby is dead. Can we just please get it out?" P5*
1D: "An awful, awful time": Devastation and subsequent pregnancies	*"Each miscarriage kind of gets you in a slightly different way and, and that kind of physical fear and post traumatic thing, you know, I even had an elective caesarean section with my [living] baby." P6*

	"I would still say that anxiety I didn't have before is still there after five years, that, at times of stress, at times of worry, it definitely creeps up. Especially in the subsequent pregnancy. [...] It was an awful, awful time". P2
**2: Loss of Baby**	
2A: 'I knew what was coming': Anticipated loss	*"So by the third time I was going to early pregnancy clinic when it was the same midwife who was scanning me again, I was convinced that just by previous experience that probably is going to be bad news." P2*<br><br>*"The second time I was an absolute pieces because I knew what was coming." P2*
2B: 'What we could have had': Grieving the loss of future	*"So I found it really difficult and obviously you know seeing the little baby and, you know, I mean, it's really tiny, but it was, you could see little arms or legs you know or what was the beginnings of those. So that was really, really quite difficult." P10*<br><br>*"So coming up to the due date when I'd see wee babies and like for a wee bit afterwards, a few weeks afterwards. I didn't, I didn't like it. I wasn't angry at those moms or wasn't jealous. But like I felt a little bit of sadness like that my child could have been like, [...] like that, it's just sadness. And yeah, what we could have had." P4*<br>*"It's not that those the other pregnancies mean any less to me, but I think because we heard the heartbeat for the one in 2017, it just made it more real or made it, you know, I don't know. I just, it just felt more significant to me." P9*<br><br>*"The first time I didn't cry very much [...] But the second time, every time she [the doctor] spoke to me I cried." P7*<br><br>*"But whenever you get positive pregnancy test, I felt like I was having a baby and that's what and all those miscarriages are. I still feel like that, you know. I have one child who thankfully is here. But there are others who aren't, and they're not, they're not any less, you know, they are my babies." P10*
2C: "As if it didn't exist": The pain of intangible loss	*"We hadn't told anybody I was pregnant. So, it was just like as if it didn't exist nearly, only to me and my husband. [...] If your child is lost later in pregnancy or stillborn, people talk about their name. Not always, but they have a name, they have an identity, they have an existence." P2*<br><br>*"Obviously a lot of people don't know about it, or they don't know that you know you were pregnant, and because there's no baby [...] it feels sometimes like, you know, those pregnancies didn't exist, or like those babies didn't exist." P10*<br><br>*"I think it made my grief worse. Just think, because it just felt like nobody understood, my grief. And it's just like, almost like normal. Get on with it. That was hard." P3*<br><br>*"You wouldn't say that ['get on with it'] to somebody that had just lost a person, a tangible being, and I think people find that hard*

	when there hasn't been a tangible baby or child there. They don't really get the grief is the same." P3<br><br>*"My language was very much about 'this is my child. I am pregnant with a baby'. But their language was 'we see nothing'. There was no mention of heartbeat. There's no mention of baby." P2*<br><br>*"You're sitting with the machine where you're supposed to be watching your baby's heartbeat. The problem is they also turn to screen away from you when it's bad news, so you can't even see it which (pause) I find hard." P1*<br><br>*"[I said] I'm having a miscarriage and unfortunately I am losing my baby". And he [the doctor] turned and said 'Ohh, that's terrible, terrible. Look at the bright side. At least you're able to get pregnant.' And I was stunned. I thought it was horrific." P3*
**3: Isolation and Guilt**	
3A: "My fault": Guilt and RPL	*"You know, I drank this, I exerted myself too much. I must have. I didn't, you know, wipe myself properly. My personal hygiene is not clean enough." P8*<br><br>*"I said no and I wanted to go [for treatment rather than waiting], but it was primarily to get a test that I think at that stage I wanted to get a test to prove that it wasn't my fault." P1*<br><br>*"When you have a reason, it's easier to process it in your head and you have an understanding of why something like that would happen and you can make your peace with it, but when you don't know it's kind of hard to do that." P10*<br><br>*"She was very cold and she said and I said, 'Oh my God, is that because I was taking tamoxifen?' She says 'we don't know it could have been anything it could have been natural'. I felt like it was my fault." P1*<br><br>*"I never actually let myself call them babies, or think about them in that way and, and it annoyed me actually when she [the midwife] tried to 'cause I think it highlighted that I [participant emphasises the word 'I'] had lost it." P6*
3B: "Don't talk about it": Isolated by miscarriage stigma	*"And you're always talking to your friends about your grief and you kinda think 'Jesus are they dreading seeing me?'" P3*<br><br>*"And when you're told, you know, it's very common, it happens to one in four, or one in three, or whatever the statistic is. That actually made me feel worse for being upset about it." P4*<br><br>*"Miscarriages, it's just so hush and taboo and people just don't talk about it." P2*
3C: "We were just caught adrift": Isolated by inadequate healthcare provision	*"There's no checking in on your welfare or your mental health. At all. [...] You're expecting a letter and when nothing comes through you feel very isolated" P2*

	"I think even just something to direct me, I don't know, not necessarily someone to talk to, but maybe even like a website or something that you could go and look at." P7<br><br>*"It does annoy me to think that there's not an awful lot of support there, you know, I felt very alone." P5*<br><br>*"I felt very alone and I felt very much like empty and numb and I think it would be useful to have access to someone who I could have talked to about those emotions and those feelings. I didn't have that." P4*<br><br>*"Prior to leaving [the hospital] they had said they would get in touch that evening just to make sure I was okay and things were going well but no contact was ever made." P6*<br><br>*"We were just caught adrift. There was no emotional support, there was no follow up" P3*<br><br>*"He [husband] felt that nobody at any stage said to him, and you know, how do you feel? Or do you need any help? Can I talk to you? So he was trying to support me through this, but nobody was actually helping him up." P9*<br><br>*"And there was never nothing offered for him. Nothing. At least I got two phone calls from the bereavement midwife. My husband got nothing, absolutely zippo." P5*
3D: 'Did you not get a leaflet the last time?": Isolated during RPL	*"The support just wasn't there. It was nearly like 'you know what you're doing'. And now you know. I think it should be explained to you like it's the first time it's happened to you every time, 'cause it's amazing how different each miscarriage is." P1*<br><br>*"I asked 'what happens? Is there anything that I can get?' and quote unquote [the nurse said] "Did you not get a leaflet the last time?". And I said, 'I did, but I didn't keep it.'" P2*
**4: Wellbeing Supports for Recurrent Miscarriage**	
4A: "They couldn't have done enough for you": Compassionate healthcare	*"The midwives were amazing. Like they were so nice. So comforting. They couldn't have done enough for you." P5*<br><br>*"But I thought there was that sort of genuine emotion behind her I felt like she wasn't just doing her job. I thought she was human, more human about it." P4*<br><br>*"And I just felt and I felt because I got that support from the her, I felt slightly more equipped to deal with it." P6*<br><br>*"If somebody is really nice to me and I feel really supported by someone, I cry. Because I feel like I can." P7*
4B: "It certainly helped me": Mental health support	*"I had eight sessions [of CBT] and I, till this day, would still use the techniques that I was taught [...] It certainly helped me." P2*

	"I actually gained a lot from it because it was, uhm, like a self-help group and ah great because I felt like I was able to support the other ones so that, you know, I was able to give something back, that it wasn't in vain. And it helps to come to terms with it so much more." P1
4C: "Sometimes I think I wouldn't be here": Social Support	*"If I hadn't had my daughter as my safe space. I probably would have been in an even darker place because I knew you had to be there for her." P2*<br><br>*"But now I have a grand- daughter and without her honestly, sometimes I think I wouldn't be here." P5*<br><br>*"I ended up just like thinking about people who I knew who had similar experiences and reaching out to them." P10*<br><br>*"Peer support whenever other people are still grieving and aren't in a healing place, is not good." P2*
4D: "Physical things to remember": Objects of loss and memory making	*"I like to have physical things to remember. You know what has happened and to remember that they were part of my life and that they are part of my life and that they exist for me. You know that they're not just something that you forget about." P10*<br><br>*"I asked for pictures each time because it's like [...] it's your first and your last picture." P1*<br><br>*"I would love to have had something to go along with my two daughters birth certificates, that there was something about their two siblings, a piece of paper to acknowledge, you know." P2*<br><br>*"I didn't really have anything to hold on to for the memory box in that way you know I didn't have like any reminders or anything like that. So it is something that I would have done if I had but I don't have those unfortunately." P3*
4E: "Wouldn't let myself believe" and "You have to make your peace with it": Coping strategies and resilience following RPL	*"I am very detached with this pregnancy. Not wanting to get my hopes up. [...] We didn't tell our family until I was 18 weeks pregnant". P4*<br><br>*"So I wouldn't, couldn't believe that I was getting a baby home with me and we had no cot, we had no clothes, we had no anything in the house. I just wouldn't let myself believe that it was all gonna work out." P6*<br><br>*"I think with the first one there's more of an emotional bond and attachment because you know, it's the first time getting pregnant, and I can remember more details from that then I actually can the others because I think you start blocking details out whenever it keeps going on." P9*

	"And then in my subsequent pregnancy and they told me I could come down at any moment and be scanned and sometimes I went down twice 'cause I was just like, oh, I'm so worried." P1<br><br>*"I'm not too sure of how many people avail of supports they and have early losses and things like that. And I just do now. As I've said it, I wonder if I had kind of been pushed into addressing things a little earlier. I might have coped a little bit better." P6*<br><br>*"Not that any of them where easy, but at least you're a little bit more prepared and, you know, whenever, you've, kind of, had that experience before." P10*<br><br>*"...would I risk another baby die? Watching another baby die with my own eyes. So I had to make the decision that I'm, I'm not gonna have a family." P8*<br><br>*"At some point you have to make your peace with it because I would have loved to have had other children and I would love for my wee girl to have a sibling but you can't, you know. It's very hard to just keep chasing something that doesn't happen because it does take a toll on you." P10*
